# Effect of You-Gui Yin on the Activities of Seven Cytochrome P450 Isozymes in Rats

**DOI:** 10.1155/2020/9784946

**Published:** 2020-05-13

**Authors:** Fan He, Ting Jiang, Shizhong Hong, Lei Wang, Weidong Chen, Li Liu

**Affiliations:** ^1^School of Pharmacy, Anhui University of Chinese Medicine, Hefei, Anhui, China; ^2^Institute of Drug Metabolism, Anhui University of Chinese Medicine, Hefei, Anhui, China; ^3^School of Medical Economics and Management, Anhui University of Chinese Medicine, Hefei, Anhui, China; ^4^Anhui Province Key Laboratory of Chinese Medicinal Formula, Hefei, Anhui, China; ^5^Synergetic Innovation Center of Anhui Authentic Chinese Medicine Quality Improvement, Hefei, Anhui, China; ^6^Center of Drug Metabolism and Pharmacokinetics, School of Pharmacy, China Pharmaceutical University, Nanjing, Jiangsu, China

## Abstract

You-Gui Yin (YGY) is a traditional Chinese medicine (TCM) decoction composed of eight Chinese herbs. The interaction between TCM and Western medicine has attracted much attention nowadays. It is therefore necessary to study the clinical application of YGY in combination with Western medicine from the perspective of metabolic enzymes. This study aims to investigate the effect of YGY on the activities of seven CYP450 isozymes (CYP1A2, CYP2B6, CYP2C8, CYP2C9, CYP2C19, CYP2D6, and CYP3A4) in rats. Twenty-four Sprague-Dawley (SD) rats were randomly divided into four groups: high, middle, and low-dose YGY-treated groups and the control group. They were given 13.78, 20.67, and 31 g/kg/d YGY decoction by oral administration and normal saline (10 mL/kg), respectively, for 14 days. Half an hour after the last administration, a mixed probe substrate (1 mg/kg) was administered by tail vein injection. Then, blood was taken from the venous plexus at different time points. The protein expression level of the CYP450 enzymes in the control and treatment groups was determined by western blot. The effect of YGY on the activity of CYP isoenzymes was studied by comparing the plasma pharmacokinetics between the control and treatment groups. Compared with the control group, YGY at a high (31 g/kg) dosage could decrease AUC_(0–*t*)_, AUC_(0–∞)_ and *C*_max_ of diclofenac, omeprazole, and midazolam by at least 35.4%, while increase CL by at least 88.9%; this revealed that YGY could induce CYP2C9, CYP2C19, and CYP3A4. The results show that when we use You-Gui Yin decoction in combination with other drugs, especially drugs metabolized by CYP2C9, CYP2C19, and CYP3A4 enzymes, the interaction between drugs needs special attention.

## 1. Introduction

The combination of traditional Chinese medicine (TCM) and Western drugs is becoming common in clinical applications leading to herb-drug interactions (HDIs) [1]. The HDIs may enhance or weaken the efficacy of the herbs. For example, there are serious herbal interactions between warfarin and pomegranate peel or guava leaf extract. Guava and warfarin may increase the risk of bleeding [2]. Clinical and animal experiments showed that *Salvia miltiorrhiza* could significantly reduce the absorption of rosuvastatin [3]. This interaction occurs at least in the absorption phase of the small intestine. However, many studies have shown that herbal medicines can affect drug metabolism [4–7].

Metabolic interactions are mainly due to the induction or inhibition of metabolic enzymes by drugs where CYP450 monooxygenases play a leading role [8]. CYP450 is the most important family of liver microsomal mixed-function oxidases [9], which participates in the metabolism of most drugs and endogenous substances *in vivo*. CYP1A2, CYP2B6, CYP2C8, CYP2C9, CYP2C19, CYP2D6, and CYP3A4 are the most important subtypes, accounting for more than 80% of the total CYP450 enzymes in the liver [10]. More than 90% of the drugs available in the market are metabolized by these seven subtypes [11]. Presently, the most widely used method to study CYP450 enzyme activity at the isozyme level in liver microsomes was first proposed by Breimer and Schellens in the late 1980s. It is referred to as the cocktail probe substrate method which is a kind of application that simultaneously gives a variety of relatively low doses of probe drugs. And it can use modern instrumental analysis technology to determine the metabolic rate or metabolic typing index of the probe drugs in biological samples to obtain multiple CYP450 isoenzyme phenotypes' information. Studying the effect of drugs on CYP activity is helpful for the rational selection of therapeutic drugs and avoiding the occurrence of toxic reactions [9]. The cocktail probe drug method agrees with the overall dialectical view of TCM in evaluating the properties of metabolites, drug interactions, phenotypic analysis of drug metabolism, and evaluation of clinical administration schemes of TCM in the liver CYP450 enzyme system. Therefore, its application value in the modernization of TCM cannot be ignored. In fact, accurate and high throughput methods can evaluate CYP450 enzyme activity [12].

YGY comes from “Jing Yue Quanshu,” which was documented by Jiebin Zhang in Ming Dynasty. It consists of Rehmanniae Radix Praeparata, Dioscoreae Rhizoma, Lycii Fructus, Corni fructus, Glycyrrhizae Radix et Rhizoma, Eucommiae Cortex, Cinnamomi Cortex, and Aconti Lateralis Radix Praeparata. The fingerprints of You-Gui Yin were studied in the domestic literature [13], and the contents of nine index components (geniposidic acid, morroniside, chlorogenic acid, geniposide, loganin, pinoresinol diglucoside, liquiritin, rutin, and glycyrrhizic acid) were determined at the same time. The quality faction of nine components were 87.6∼119.1 *μ*g/g, 323.6∼365.6 *μ*g/g, 108.3∼124.1 *μ*g/g, 79.5∼85.0 *μ*g/g, 171.7∼188.0 *μ*g/g, 163.0∼238.3 *μ*g/g, 64.5∼53.3 *μ*g/g, 159.8∼168.5 *μ*g/g, and 72.8∼83.6 *μ*g/g. YGY has been widely used in combination therapy for knee osteoarthritis, early steroid-induced femoral head necrosis, and refractory asthma. YGY combined with *Staphylococcus aureus* can treat early steroid-induced femoral head necrosis [14]. And the therapeutic effect of YGY combined with hormone drugs on refractory asthma is remarkable [15]. However, research on the mechanism of action of TCM is still not systematic and comprehensive enough and has been attracting the attention of researchers in many countries. Although the components of TCM are complex, the basis of its pharmacodynamics mostly depends on the metabolism of drug metabolizing enzymes or on the inhibition or induction of drug metabolizing enzymes [16], thus affecting the metabolism of other drugs or drug interactions, which interferes with the effectiveness and safety of drugs.

The aim of this study was to investigate the effect of YGY on the activities of seven hepatic CYP450 isozymes by comparing the plasma pharmacokinetics of phenacetin, bupropion, amodiaquine, diclofenac, omeprazole, dextromethorphan, and midazolam in rats. Pharmacokinetic parameters were compared between the control group and the YGY administration group. This work provides a theoretical guidance for the safe clinical use of YGY on patients and helps to promote further development of YGY in the treatment of orthopedic and asthmatic diseases.

## 2. Materials and Methods

### 2.1. Chemicals and Reagents

Decoction pieces of Rehmanniae Radix Praeparata (*Rehmannia glutinosa* Libosch.), Dioscoreae Rhizoma (*Dioscorea opposita* Thunb.), Lycii Fructus (*Lycium barbarum* L.), Corni fructus (*Cornus officinalis* Sieb. et Zucc.), Glycyrrhizae Radix et Rhizoma (*Glycyrrhiza uralensis* Fisch.), Eucommiae Cortex (*Eucommia ulmoides* Oliv.), Cinnamomi Cortex (*Cinnamomum cassia* Presl.), and Aconti Lateralis Radix Praeparata (*Aconitum carmichaeli* Debx.) were obtained from Anhui Puren Traditional Chinese Medicine Pieces Co., Ltd. (Hefei, China). All the above herbs were produced from Henan Province. All medicinal herbs were identified by Prof. Nianjun Yu of Anhui University of Traditional Chinese Medicine.

Phenacetin, bupropion, amodiaquine, diclofenac, omeprazole, dextromethorphan, midazolam (purity >98%), and the internal standard (IS) glibenclamide were purchased from the National Institute for Food and Drug Control (Beijing, China). Acetonitrile and methanol were chromatographically purified, while other reagents were of analytical grade.

### 2.2. Preparation of YGY Decoction

The following eight Chinese herbs were soaked in water for half an hour, Rehmanniae Radix Praeparata (9 g), Dioscoreae Rhizoma (6 g), Lycii Fructus (6 g), Glycyrrhizae Radix et Rhizoma (9 g), Corni fructus (5 g), Eucommiae Cortex (3 g), Cinnamomi Cortex (6 g), and Aconti Lateralis Radix Praeparata (6 g). The herbs were then decocted 2 times with water, the residues were discarded, and the mixed decoction was concentrated to 1.5 g/mL, then stored at 4°C.

### 2.3. Animals

SD rats (male, 220 ± 20 g) were purchased from the Animal Laboratory Center of Anhui Medical University (Hefei, China), certificate number SCXK (wan) 2017-001. The rats were housed under natural light-dark cycle conditions with controlled temperature (25°C) and humidity (60 ± 5%). The experiment was commenced one week after adaptive feeding. All experimental procedures were ethically approved by the Administration Committee of Experimental Animals of Anhui University of Chinese Medicine.

### 2.4. Plasma Pharmacokinetics

Twenty-four SD rats (male, 220 ± 20 g) were randomly divided into four groups: control group (CG), low-dose YGY group (LG), middle-dose YGY group (MG), and high-dose YGY group (HG). CG was administered with saline (10 mL/kg). The YGY group was orally administered with 13.78 g/kg/d of LG, 20.67 g/kg/d of MG, and 31 g/kg/d of HG for 2 consecutive weeks. After the last dose, the control group and the YGY group were administered with mixed probe solution (phenacetin, bupropion, amodiaquine, biclofenac, omeprazole, dextromethorphan, and midazolam of 1 mg/kg) by tail vein injection. Blood samples (about 0.25 mL) were taken at 0.05, 0.083, 0.167, 0.25, 0.5, 0.75, 1, 2, 4, 8, and 12 h after administration. The mixed probe substrate was dissolved by methanol. The plasma samples were separated by centrifuging at 3500 g for 15 min and then stored at −80°C.

### 2.5. Plasma Sample Preparation

After transferring the plasma sample (90 *μ*L) into a 1.5 mL centrifuge tube, 10 *μ*L mixed probe solution and 500 ng/mL glibenclamide solution (dissolved by methanol) 10 *μ*L were added and 300 *μ*L of acetonitrile was finally added into the tube for protein precipitation. After mixing the sample vortices for 3 min, they were centrifuged at a speed of 3500 rpm/min for 10 min. 2 *μ*L of the supernatant was injected into the UPLC-MS system for analysis.

### 2.6. Western Blot Analysis

Twelve male SD rats were randomly divided into four groups, three in each group. They were fed under the same conditions as the above mentioned 24 rats. On the 14th day, the rats were killed by bleeding from the abdominal aorta after intragastric administration. The liver tissues were taken out quickly, washed, and stored in a refrigerator at −80°C. All groups of protein were tested by bicinchoninic acid (BCA) assays (Beyotime, Jiangsu, China). The protein was loaded on 10% sodium dodecyl sulfate-polyacrylamide gel electrophoresis (SDS-PAGE). Then, the proteins were shifted to a nitrocellulose filter membrane (NC) after SDS-PAGE. Tris-buffered saline and 1% Tween 20 (TBST) were added to normal saline with 5% skim milk, and the nonspecific protein was blocked for 1.5 h. Then, it was immersed at 4°C using primary antibodies: CYP2C9 (#DF10127, Affinity, Dilution: 1 : 1000), CYP2C19 (#AF0744, Affinity, Dilution: 1 : 1000), CYP3A4 (#DF7001, Affinity, Dilution: 1 : 1000), and *β*-actin (TA-09, ZSGB-BIO, Beijing, China) overnight at 4°C. Secondary antibodies peroxidase-conjugated goat antimouse IgG (H + L) and peroxidase-conjugated goat antirabbit IgG (H + L) were purchased from ZSGB-BIO (Beijing, China). Digital images of protein bands were collected by Chemidoc XRS (BioRad).

### 2.7. Statistical Analysis

The pharmacokinetic parameters of phenacetin, bupropion, amodiaquine, diclofenac, omeprazole, dextromethorphan, midazolam, AUC, MRT, *T*_1/2_, and CL were calculated by DAS 2.0 software (Chinese Pharmacological Association, Beijing, China) according to the blood concentration of probes at different time points. Statistical analysis was performed by SPSS 21.0 software (SPSS Inc., Chicago, IL, USA), and the pharmacokinetic parameters of the experimental group and the blank control group were analyzed by the *t*-test. A difference was deemed statistically significant when *p* < 0.05.

## 3. Results

### 3.1. Method Validation

#### 3.1.1. Specificity

**Figure 1:**
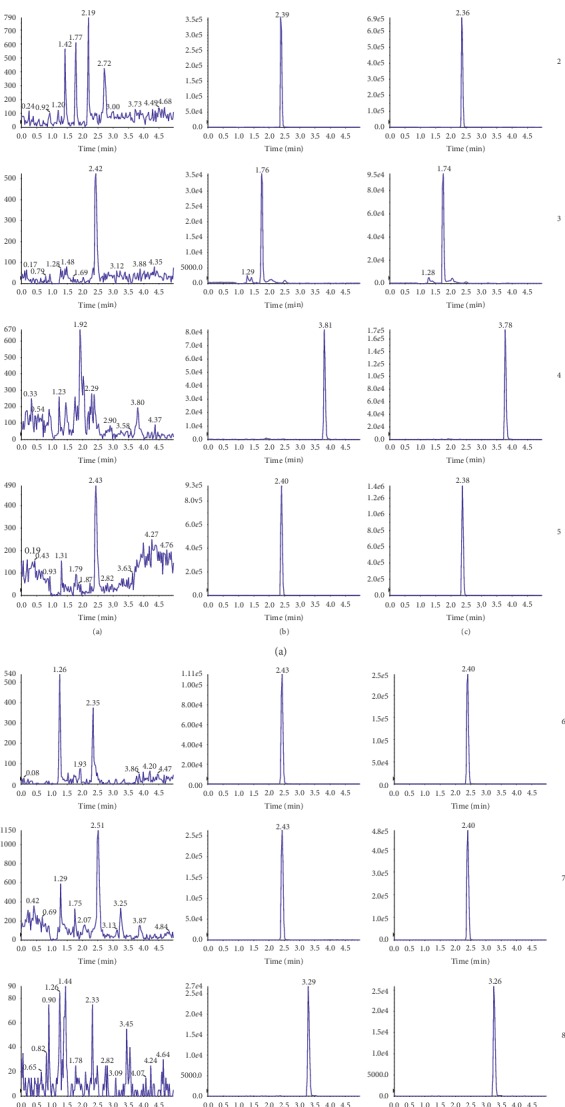
UPLC-MS/MS chromatograms of probes and the internal standard in SD rat plasma. (a) Blank plasma; (b) blank plasma with mixed probe substrates and glibenclamide (IS); (c) plasma sample obtained from a rat after intravenous injection of the cocktail probe drugs spiked with the IS; (1) phenacetin; (2) bupropion; (3) amodiaquine; (4) diclofenac; (5) omeprazole; (6) dextromethorphan; (7) midazolam; and (8) glibenclamide.

As shown in Figure 1, probe drugs and the internal standard were not affected by the endogenous substances in rat blank plasma, and the effects of adding mixed probes in rat blank plasma were consistent with those of probes in plasma after administration. Therefore, the UPLC-MS/MS method for simultaneous determination of seven probes in rat plasma had good selectivity and specificity.

#### 3.1.2. Calibration Curve and Sensitivity

The standard curves and linear ranges of probe substrates in plasma are shown in Table 1.

#### 3.1.3. Interday and Intraday Precision and Stability

The results of interday and intraday precision of seven probe drugs are shown in Table 2. The results showed that inter and intraday precision ranged from 0.13% to 12.3%. In the stability experiment, we investigated the short-term stability and long-term stability of each probe substrate, including room temperature stability, automatic injector stability, and repeated freeze-thaw stability. Long-term stability refers to the stability of plasma samples stored in a −20°C refrigerator for 7 days. The results show that the RSD value of all quality control samples is between 1.43% and 13.51%, meeting the requirements of biological sample analysis.

### 3.2. Pharmacokinetic Parameters of Probe Substrates in Rats

#### 3.2.1. Effects of YGY on the Activity of CYP1A2 in Rats (*n* = 6)

**Figure 2:**
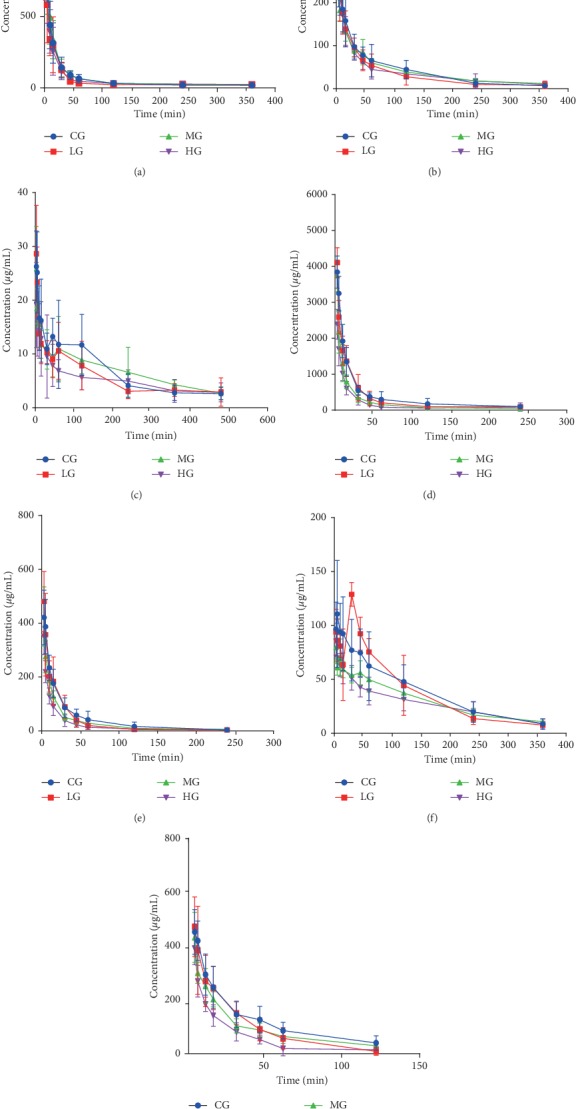
Pharmacokinetic curves of probe substrates in rats. (a) Phenacetin; (b) bupropion; (c) amodiaquine; (d) diclofenac; (e) omeprazole; (f) dextromethorphan; and (g) midazolam.

Phenacetin pharmacokinetic profiles were presented in the study groups to describe CYP1A2 activity. The pharmacokinetic profiles and mean plasma concentration-time curves of phenacetin in rats are presented in Table 3 and Figure 2. Compared with the CG, the main pharmacokinetic parameters AUC_(0–*t*)_ and AUC_(0–∞)_ in LG decreased significantly by 46.2% and 48.8%, but the CL did not change significantly in all groups. It can therefore be inferred that YGY had no significant effect on CYP1A2 in rats.

#### 3.2.2. Effects of YGY on the Activity of CYP2B6 in Rats (*n* = 6)

Pharmacokinetic profiles of bupropion after YGY administration were used to present the activity of CYP2B6. The main parameters are shown in Table 3. The mean plasma concentration-time curves of bupropion in the control and YGY groups are shown in Figure 2. The main pharmacokinetic parameters AUC_(0–*t*)_, AUC_(0–∞)_, and CL of the YGY group did not change significantly compared to those of the control group, indicating that YGY did not significantly affect CYP2B6 in rats after two weeks of continuous administration.

#### 3.2.3. Effects of YGY on the Activity of CYP2C8 in Rats (*n* = 6)

CYP2C8 activity was determined by comparing pharmacokinetic profiles of amodiaquine between the control group and the YGY group. The effects of YGY on pharmacokinetic parameters and the mean plasma concentration-time curves of amodiaquine before and after oral administration of YGY for two weeks are shown in Table 3 and Figure 2. The main pharmacokinetic parameters AUC_(0–*t*)_, AUC_(0–∞)_, and CL of the YGY dose group had no significant difference compared to those of the control group. These results indicate that YGY groups had no significant effect on CYP2C8 in rats.

#### 3.2.4. Effects of YGY on the Activity of CYP2C9 in Rats (*n* = 6)

Diclofenac is a specific probe substrate for CYP2C9 in rats. The effect of YGY on the pharmacokinetic parameters of phenacetin in rats is shown in Table 4. The mean plasma concentration-time curve of phenacetin in the control group and YGY group is shown in Figure 2. Compared with CG, the main pharmacokinetic parameters AUC_(0–*t*)_, AUC_(0–∞)_, and *C*_max_ in the high-dose group decreased significantly by 50.3%, 52.8%, and 47.5%, respectively, while the CL of diclofenac increased significantly by 109.1%, indicating that CYP2C9 activity was induced by a high dose of YGY in rats.

#### 3.2.5. Effects of YGY on the Activity of CYP2C19 in Rats (*n* = 6)

Omeprazole was used to evaluate the activity of CYP2C19 in rats. The effect of YGY on pharmacokinetic parameters of omeprazole in rats is depicted in Table 4. The corresponding pharmacokinetic parameters of omeprazole are shown in Figure 2. Compared with CG, the main pharmacokinetic parameters AUC_(0–*t*)_, AUC_(0–∞)_, and *C*_max_ in HG decreased significantly by 50.3%, 47.6%, and 43.1%, significantly, while CL in HG increased significantly by 88.9%. Based on these findings, it can be concluded that high-dose YGY could induce CYP2C19 in rats.

#### 3.2.6. Effects of YGY on the Activity of CYP2D6 in Rats (*n* = 6)

The activity of CYP2D6 was evaluated by analyzing the pharmacokinetic parameters of dextromethorphan in rats after two consecutive weeks of administration. As shown in Table 4 and Figure 2, no significant changes were observed on the pharmacokinetic parameters AUC_(0–*t*)_, AUC_(0–∞)_, and CL of omeprazole in the treatment groups compared with the blank group. These results suggest that YGY had no influence on the activity of CYP2D6 in rats.

#### 3.2.7. Effects of YGY on the Activity of CYP3A4 in Rats (*n* = 6)

Midazolam is the most suitable substrate for CYP3A4 and is usually used to express the activity of CYP3A4. The main parameters are shown in Table 4 while the mean plasma concentration-time curves of midazolam in CG and YGY groups are shown in Figure 2. Compared with CG, AUC_(0–*t*)_, AUC_(0–∞)_, and *C*_max_ of midazolam pharmacokinetic parameters in HG were significantly decreased by 48.2%, 52.6%, and 35.4%, respectively, and CL was significantly increased by 103.6%, which indicated that a high dose of YGY for two consecutive weeks might induce CYP3A4 in rats.

### 3.3. Effects of YGY on CYP2C9, CYP2C19, and CYP3A4 Protein Expression in Rats (*n* = 3)

**Figure 3:**
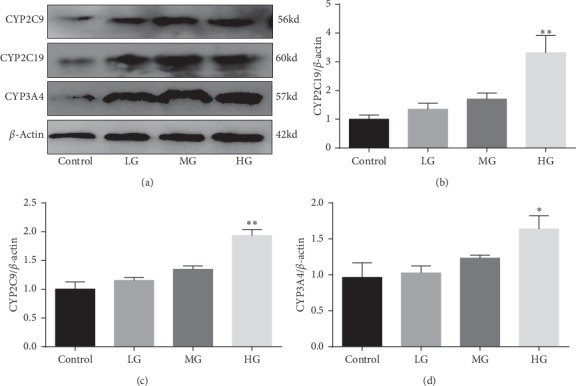
Effects of YGY on CYP2C9, CYP2C19, and CYP3A4 protein expression in rats. Values are mean ± SD of 3 independent experiments. ^*∗*^*P*  <  0.05 and ^*∗∗*^*P*  <  0.01, compared with the control group.

The expression of CYP2C9, CYP2C19, and CYP3A4 in rats treated with YGY was further verified via western blotting. As shown in Figure 3, compared with the control group, the expression of CYP2C9, CYP2C19, and CYP3A4 in the HG was significantly increased (*P* < 0.01). These results were consistent with those in previous *in vivo* experiments.

## 4. Discussion

CYP1A2 mainly exists in the liver, it accounts for about 13% of the total human CYPs, and about 20% of commonly used clinical drugs are metabolized by CYP1A2 [17]. CYP1A2 is also involved in the metabolism of some antipyretic and analgesic drugs, such as paracetamol and naproxen. Based on the results of this study, YGY had no significant effect on CYP1A2 in rats, it was presumed that the interaction between YGY and drugs metabolized by CYP1A2 was unlikely.

CYP2B6 represents about 1–10% of the total hepatic CYPs. It is well known that this enzyme can metabolize some exogenous substances, such as anticancer drugs cyclophosphamide and interferon. In addition, the expression and function of the human CYP2B6 gene has significant interindividual variations, which may lead to changes in clinical outcomes in patients receiving CYP2B6 substrates. These differences come from many sources, including genetic polymorphism and exogenous intervention [18]. In this study, it was found that CYP2B6 activities were not significantly affected by YGY. Based on this, YGY can be used in diverse clinical applications alongside drugs metabolized by CYP2B6, but individual differences should also be considered.

The CYP2C subfamily plays a fatal role in the metabolism of many drugs including toluene sulfonylurea, S-warfarin [19, 20], phenytoin [21], citalopram [22] and thalidomide, CYP2C8, CYP2C9, and CYP2C19 were significantly expressed in the adult liver, but the expression characteristics of the CYP2C subfamily in extrahepatic tissues (such as the brain) were not obvious [23]. The current research shows that YGY had no effect on CYP2C8 in rats, but a high dose of YGY induced CYP2C9 and CYP2C19 and significantly enhanced the protein expression level to some extent, which suggests that when YGY was combined with other drugs metabolized by CYP2C9 and CYP2C19, the blood concentration of drugs decreased and the metabolic rate increased, which needs to be explored further.

Although CYP2D6 only accounts for <2% of the total CYPs in the human liver [24], it participates in 25% of drug metabolism in the market [25]. The commonly used antidepressants such as desipramine, nortriptyline, and paroxetine, the antipsychotic drug perphenazine, and the antihypertensive indolamine are metabolized by CYP2D6. These results clarified that YGY had no effects on the CYP2D6 in rats. It is suggested that when YGY is used in combination with other medications, there may be no need to worry about the drug interaction.

CYP3A4 accounts for about 30% of the total CYP enzymes in the human liver and participates in the metabolism of more than 50% of clinical drugs [26]. It is involved in the transformation of more than 150 drugs into 38 categories, including 6-hydroxylation and 16-hydroxylation of endogenous hormones and the activation of some precancerous substances [27]. In this study, the high-dose group of YGY was found to potentially induce the CYP3A4 enzyme, while the protein expression level of CYP3A4 also increased significantly in the high-dose group. This indicates that YGY was combined with drugs metabolized by CYP3A4, while HDIs should be analyzed to prevent adverse reactions.

## 5. Conclusion

In this study, a highly specific and sensitive UPLC-MS/MS method was used to determine the concentration of seven probe substrates in rat plasma. The method had good accuracy, precision, and stability and was successfully applied to evaluate the activity of seven metabolic enzymes (CYP1A2, CYP2B6, CYP2C8, CYP2C9, CYP2C19, CYP2D6, and CYP3A4) in rats. The results of this study provide some useful information for the clinical application of YGY alone or in combination with other drugs. But this information still needs to be validated by clinical trials or in vitro experiments.

## Figures and Tables

**Table 1:** Liner range, regression equation, and correlation coefficient for seven probe drugs.

Compounds	Liner range (ng/mL)	Regression equation	Correlation coefficient (*r*)
Phenacetin	10∼1500	*y* = 0.0794* *− 1.473	0.9988
Bupropion	0.25∼400	*y* = 0.0497*x *− 0.0635	0.9994
Amodiaquine	0.25∼50	*y* = 0.1103*x *− 0.0441	0.9960
Diclofenac	20∼6400	*y* = 0.0015*x *− 0.0084	0.9997
Omeprazole	2∼750	*y* = 0.1054*x *− 0.2935	0.9981
Dextromethorphan	0.5∼150	*y* = 0.0466*x *− 0.0672	0.9980
Midazolam	1∼640	*y* = 0.0362*x* + 0.1179	0.9972

**Table 2:** Interday and intraday precision of seven probe drugs in rat plasma.

	Add (ng/mL)	Interday precision		Intraday precision	
		Mean ± SD (ng/mL)	RSD (%)	Mean ± SD (ng/mL)	RSD (%)
Phenacetin	20	20.43 ± 1.72	8.44	19.91 ± 1.20	6.03
	50	52.19 ± 0.93	1.78	49.61 ± 2.02	4.07
	1200	1208.04 ± 40.04	3.31	1220.00 ± 5.32	0.43

Bupropion	0.5	0.50 ± 0.05	11.57	0.52 ± 0.03	7.66
	25	24.67 ± 0.32	1.30	25.31 ± 0.61	2.42
	320	324.07 ± 12.12	3.74	321.83 ± 3.44	1.06

Diclofenac	40	39.21 ± 0.73	1.86	39.38 ± 1.06	2.71
	1000	1025.65 ± 16.92	1.65	1010.52 ± 19.73	1.95
	5120	4933.36 ± 100.09	2.02	5110.28 ± 40.97	0.80

Amodiaquine\	0.5	0.52 ± 0.02	4.69	0.52 ± 0.03	7.28
	10	9.72 ± 0.51	5.31	10.82 ± 0.37	3.46
	40	39.35 ± 2.04	5.20	42.77 ± 1.23	2.88

Omeprazole	4	3.84 ± 0.47	12.30	4.06 ± 0.18	4.61
	100	101.09 ± 4.28	4.23	97.78 ± 1.44	1.47
	600	595.17 ± 11.55	1.94	606.19 ± 18.86	3.11

Dextromethorphan	1	0.98 ± 0.08	8.49	0.53 ± 0.04	8.44
	20	20.51 ± 0.59	2.89	21.00 ± 0.40	1.91
	120	118.30 ± 3.15	2.66	122.14 ± 1.17	0.90

Midazolam	2	1.97 ± 0.15	8.01	2.00 ± 0.12	6.35
	50	51.10 ± 0.24	0.47	50.44 ± 0.67	0.13
	512	514.25 ± 15.24	2.96	514.92 ± 12.00	2.33

**Table 3:** Pharmacokinetic parameters of probe substrates in rats (*n* = 6).

CYPs	Parameters	CG	LG	MG	HG
CYP1A2	AUC_(0–*t*)_ (*μ*g/mL *∗* min)	23.76 ± 4.14	12.69 ± 3.64^*∗∗*^	23.71 ± 3.82	21.62 ± 4.24
	AUC_(0–∞)_ (*μ*g/mL *∗* min)	26.11 ± 3.05	13.37 ± 3.83^*∗∗*^	26.60 ± 4.75	54.19 ± 28.36
	*C*_max_ (*μ*g/L)	731.71 ± 130.08	651.93 ± 194.76	666.06 ± 150.93	582.09 ± 131.24
	*T*_1/2_ (min)	131.08 ± 51.17	141.84 ± 58.28	123.57 ± 117.91	120.15 ± 81.23
	CL (L/min/kg)	0.039 ± 0.004	0.04 ± 0.02	0.039 ± 0.007	0.02 ± 0.01

CYP2B6	AUC_(0–*t*)_ (*μ*g/mL *∗* min)	15.06 ± 4.18	12.88 ± 4.02	14.87 ± 1.53	12.79 ± 3.27
	AUC_(0–∞)_ (*μ*g/mL *∗* min)	16.18 ± 4.00	13.40 ± 4.25	16.69 ± 1.59	13.30 ± 3.45
	*C*_max_ (*μ*g/L)	184.38 ± 52.69	239.23 ± 60.79	190.86 ± 34.25	218.76 ± 41.79
	*T*_1/2_ (min)	97.57 ± 49.83	75.77 ± 14.62	116.10 ± 32.76	81.97 ± 10.06
	CL (L/min/kg)	0.06 ± 0.01	1.64 ± 0.60^*∗∗*^	0.06 ± 0.006	0.08 ± 0.02

CYP2C8	AUC_(0–*t*)_ (ug/mL∗min)	0.31 ± 0.07	0.27 ± 0.09	0.33 ± 0.10	0.24 ± 0.09
	AUC_(0–∞)_ (ug/mL∗min)	0.34 ± 0.08	0.37 ± 0.17	0.41 ± 0.10	0.35 ± 0.20
	*C*_max_ (*μ*g/L)	25.16 ± 7.53	23.52 ± 6.26	19.51 ± 3.20	15.92 ± 6.05^*∗*^
	*T*_1/2_ (min)	142.06 ± 54.05	252.85 ± 86.51	226.42 ± 93.44	260.97 ± 187.48
	CL (L/min/kg)	0.30 ± 0.10	0.31 ± 0.13	0.25 ± 0.07	0.37 ± 0.22

YGY groups were compared with the control group; ^*∗*^*p* < 0.05; ^*∗∗*^*p* < 0.01.

**Table 4:** Pharmacokinetic parameters of probe substrates in rats (*n* = 6).

CYPs	Parameters	CG	LG	MG	HG
CYP2C9	AUC_(0–*t*)_ (*μ*g/mL *∗* min)	88.15 ± 16.98	73.49 ± 22.70	52.89 ± 17.98^*∗*^	43.77 ± 10.14^*∗∗*^
	AUC_(0–∞)_ (*μ*g/mL *∗* min)	98.47 ± 33.79	76.70.±24.57	55.30 ± 16.80	46.47 ± 12.30^*∗∗*^
	*C*_max_ (*μ*g/L)	3249.80 ± 477.10	2886.50 ± 910.14	2168.10 ± 580.76	1704.55 ± 469.94^*∗∗*^
	*T*_1/2_ (min)	44.82 ± 32.28	33.26 ± 17.47	41.09 ± 13.74	66.95 ± 32.50^*∗∗*^
	CL (L/min/kg)	0.011 ± 0.004	0.015 ± 0.006	0.020 ± 0.006	0.023 ± 0.006^*∗*^

CYP2C19	AUC_(0–*t*)_ (*μ*g/mL *∗* min)	11.69 ± 4.26	9.33 ± 2.43	8.02 ± 1.90^*∗*^	5.81 ± 1.66^*∗∗*^
	AUC_(0–∞)_ (*μ*g/mL *∗* min)	12.00 ± 4.50	9.37 ± 2.49	8.30 ± 1.82^*∗*^	6.29 ± 1.98^*∗∗*^
	*C*_max_ (*μ*g/L)	386.85 ± 100.73	392.31 ± 99.73	187.68 ± 52.60^*∗∗*^	219.93 ± 40.77^*∗∗*^
	*T*_1/2_ (min)	46.88 ± 19.39	29.19 ± 9.09	52.10 ± 20.39	79.74 ± 61.35
	CL (L/min/kg)	0.09 ± 0.02	0.11 ± 0.03	0.12 ± 0.02	0.17 ± 0.04^*∗∗*^

CYP2D6	AUC_(0–*t*)_ (*μ*g/mL *∗* min)	13.64 ± 3.38	14.13 ± 7.42	14.96 ± 2.92	13.58 ± 3.20
	AUC_(0–∞)_ (*μ*g/mL *∗* min)	14.97 ± 3.34	15.09 ± 7.33	15.32 ± 3.01	14.91 ± 3.23
	*C*_max_ (ug/L)	116.17 ± 50.04	156.60 ± 98.86	73.13 ± 13.31	98.33 ± 30.40
	*T*_1/2_ (min)	99.92 ± 36.30	95.19 ± 25.69	98.49 ± 40.09	99.92 ± 36.30
	CL (L/min/kg)	1.40 ± 0.37	1.55 ± 0.63	1.52 ± 0.31	1.40 ± 0.37

CYP3A4	AUC_(0–*t*)_ (*μ*g/mL *∗* min)	16.09 ± 3.54	13.31 ± 4.17	12.19 ± 2.46^*∗*^	8.33 ± 2.30^*∗∗*^
	AUC_(0–∞)_ (*μ*g/mL *∗* min)	19.78 ± 7.30	13.98 ± 4.78^*∗*^	14.16 ± 4.72	9.38 ± 2.82^*∗∗*^
	*C*_max_ (*μ*g/L)	414.02 ± 71.52	407.09 ± 121.13	301.84 ± 41.80	267.62 ± 57.04^*∗*^
	*T*_1/2_ (min)	48.05 ± 33.65	25.75 ± 8.94	40.12 ± 20.57	36.02 ± 23.21
	CL (L/min/kg)	1.12 ± 0.38	1.62 ± 0.72	1.52 ± 0.42	2.28 ± 0.62^*∗*^

YGY groups were compared with the control group; ^*∗*^*p* < 0.05; ^*∗∗*^*p* < 0.01.

## Data Availability

The data used to support the findings of this study are available from the corresponding author upon request.
